# Association between Blood Vitamin D Levels and Regular Physical Activity in Korean Adolescents

**DOI:** 10.3390/healthcare10071277

**Published:** 2022-07-10

**Authors:** Jiyoun Kim, Jinho Park, Wi-Young So

**Affiliations:** 1 Department of Exercise Rehabilitation, Gachon University, Incheon 21936, Korea; eve14jiyoun@gachon.ac.kr; 2 Gachon Biomedical Convergence Institute, Gachon University Gil Medical Center, Incheon 21565, Korea; 3 Sports Medicine Major, College of Humanities and Arts, Korea National University of Transportation, Chungju-si 27469, Korea

**Keywords:** adolescents, KNHANES, physical activity, vitamin D deficiency

## Abstract

This study aimed to investigate the relationship between vitamin D levels and physical activity in adolescents using data from the Korea National Health and Nutrition Survey (KNHANES). The serum vitamin D concentrations were measured between 2008 and 2014 and analyzed. Adolescents aged 13 to 18 years comprised 4527 of the 61,370 participants surveyed. A final dataset including 2811 adolescents was obtained after those with missing data were excluded. Those who did not participate or only participated in 1–3 days of intense physical activity (*p* < 0.001), moderate physical activity (*p* = 0.002), and muscular exercise (weight training) (*p* < 0.001) showed a higher level of vitamin D deficiency than those who performed the same activities 4–7 days per week. Our results showed that most of the adolescents (76%) were vitamin D-deficient, and the risk was higher among high school than middle school students (odds ratio (OR) = 1.70, 95% confidence interval (CI) = 1.40–2.06; *p* < 0.001) and higher in girls than boys (OR = 1.43, 95% CI = 1.18–1.72; *p* < 0.001). Reduced participation in physical activity was correlated with vitamin D deficiency. Furthermore, vitamin D deficiency was associated with a lower frequency of participation in all forms of physical activity, and the association increased significantly with an increase in body mass index.

## 1. Introduction

Vitamin D deficiency is a severe global problem, so much so that the term “vitamin D deficiency pandemic” is currently being adopted [1]. A vitamin D deficit is found to cause a depressed mood [2], and an increase in vitamin D is correlated with a positive mood and a feeling of well-being [3]. Vitamin D is produced in the body following sunlight exposure and ingesting food or nutritional supplements [4]. Vitamin D circulates in the blood in the form of serum 25-hydroxyvitamin D (25(OH)D), the concentration of which is generally used to determine vitamin D levels [5]. The recommended range for the optimal serum vitamin D level (to maintain calcium homeostasis without causing hyperparathyroidism) in healthy adults is a 25(OH)D concentration of no lower than 20 ng/mL but preferably ≥30 ng/mL to maintain bone health [6]. Therefore, a serum 25(OH)D level of <20 ng/mL is generally defined as “deficient,” and a level of ≥30 ng/mL is considered “sufficient” [7,8].

Korea is one of the countries with individuals who have severe vitamin D deficiency. A large international study of menopausal women with osteoporosis conducted by Lim et al. [9] reported the rate of vitamin D deficiency in Thailand (47%), Malaysia (49%), Japan (90%), and Korea (92%) based on total 25(OH)D levels. Thus, vitamin D deficiency is considered a serious problem in Korea [10]. Vitamin D is involved in calcium and phosphorus metabolism and plays an essential role in bone development, growth, and maintenance of a healthy skeleton in adolescents. Vitamin D deficiency plays a vital role in obesity [11], diabetes [12], asthma, rhinitis [13,14], and atopic dermatitis [15,16] in adolescence. Therefore, maintaining an appropriate vitamin D level during this period is important for managing potential risk factors for future adult osteoporosis and overall health in adulthood.

Indoor and outdoor physical activity increases the plasma levels of vitamin D [17]. Physical activity contributes to increased vitamin D levels and bone mass, reduced calcium excretion, increased absorption, and an increased blood calcium level, which reduces serum vitamin D [18]. However, vitamin D deficiency results in muscle weakness, pain, and injury and increases disease frequency and duration [19,20]. A previous study on children revealed that the amount of vitamin D in the blood increases as physical activity increases, even when their exposure to sunlight is controlled [21]. In contrast, studies have also reported that the risk of vitamin D deficiency increases 1.32-fold if the duration of outdoor activity is <30 min per day [22]. Many studies on physical activity and vitamin D levels have been conducted in older people [23] and adults [24]; however, studies in adolescents are lacking. Therefore, large-scale research is warranted to confirm the relationship between physical activity and vitamin D levels in adolescents to facilitate the proactive management of adolescent health. This study aimed to investigate the relationship between vitamin D status in adolescents and physical activity related to deficiency using data from the Korea National Health and Nutrition Survey (KNHANES), a large-scale domestic survey.

## 2. Materials and Methods

### 2.1. Participants

**Figure 1:**
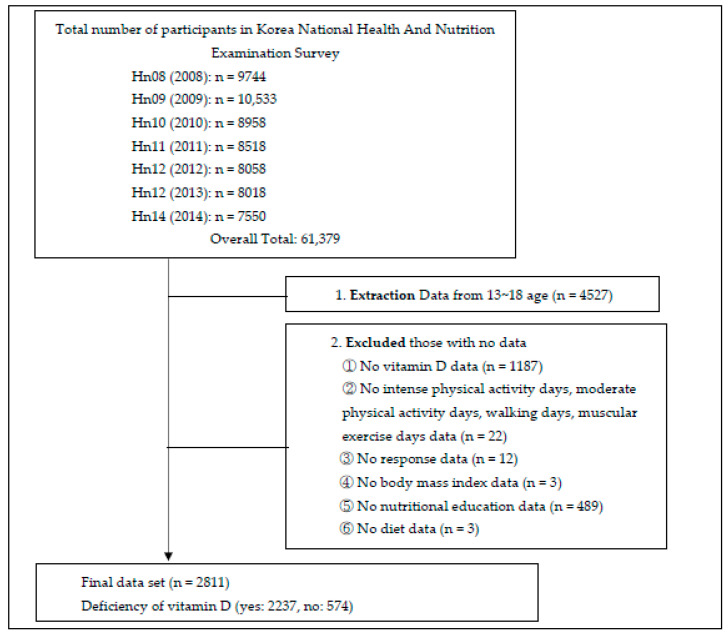
Flowchart of adolescents’ selection from the Korea National Health and Nutrition Examination Survey database.

This study used raw data from the fourth and fifth stages of the KNHANES. These data were provided and used in accordance with the Korea Centers for Disease Control and Prevention’s disclosure and management regulations and were approved by the Korea Centers for Disease Control and Prevention Research Ethics Committee (approval no. 2010-02CON-21-C, 20102CON-06-C, 2013-07CON-03-4C, 2013-12EXP-03-5C). The KNHANES consists of a health examination and nutritional surveys and is conducted to calculate basic statistics to understand health, health-related consciousness, and population behavior. Data from 2008 to 2014 on serum vitamin D concentrations were analyzed. In total, 61,379 subjects were surveyed from 2008 to 2014. Among these, adolescents aged 13–18 were classified as middle and high school students (n = 4527), of which 2811 with serum vitamin D results were finally selected (Figure 1).

### 2.2. Study Variables

#### 2.2.1. Blood Analysis: Vitamin D

Fasting blood samples were collected from all participants in the KNHANES. A serum 25(OH)D concentration of 20 ng/mL (50 nmol/L) was considered the cut-off value of insufficiency [25,26], and this criterion was adopted for this study. Therefore, a serum 25(OH)D concentration of >20 ng/mL was defined as “normal,” whereas levels <20 ng/mL were considered “deficient.”

#### 2.2.2. Physical Activity Assessment

The International Physical Activity Questionnaire was used to assess the participants’ physical activity levels. Participants were questioned regarding their level of physical activity during a typical week. They were also asked the following questions regarding moderate physical activity: “During the last seven days, how many days did you participate in moderate physical activity?” and “On these occasions, did you perform a moderate-intensity activity, e.g., tennis doubles, volleyball, badminton, table tennis, or any other activity that causes a slight increase in breathing or heart rate for at least 10 min?” In terms of intense physical activity, participants were asked: “On how many days during the past week did you do vigorous physical activity (for at least 10 min), such as running, mountain climbing, soccer, basketball, or any other activity that caused a substantial increase in breathing or heart rate?” The participants indicated the number of days per week devoted to each type of activity. They also stated the total exercise time per day with a minimum of 10 min. KNHANES physical activity levels were surveyed using the following four exercise categories: (1) intense physical activity (being out of breath due to performing the exercise for longer than 10 min); (2) medium-intensity physical activity (moderate-level physical activity, resulting in harder than usual breathing or slightly shorter than usual breaths, was performed for longer than 10 min); (3) resistance exercise (push-ups, sit-ups, dumbbells, weights, and iron bars); (4) walking exercise only (including commuting to work, commuting to and from school, and walking for exercise). The criteria were 0 days, 1–3 days, and 4–7 days. Each participant could be part of multiple exercise categories.

#### 2.2.3. Age Difference

The survey participants included adolescents aged 13–18 years with data from 2008–2014; 2811 participants with serum vitamin D results were finally selected. The participants were grouped into middle school students (13–15 years old) and high school students (16–18 years old).

#### 2.2.4. Body Mass Index

The body mass index (BMI) was measured through the health indicator examination survey of the KNHANES. BMI was calculated as weight/height (kg/m^2^) and classified as normal (18.5–22.9 kg/m^2^), underweight (<18.5 kg/m^2^), overweight (23–24.9 kg/m^2^), and obese (>25 kg/m^2^).

#### 2.2.5. Nutritional Education and Diet

Nutrition education and diet management were measured through the KNHANES health management survey. Participants were questioned about whether they had received nutritional education and counseling from public health centers, community centers, ward offices, schools, welfare facilities, or hospitals over the previous year. In addition, the diet management question was asked to ascertain whether they had experience in diet control to manage their weight from the previous year. The answers to the two surveys were confirmed as a yes or no.

### 2.3. Statistical Analyses

Data analysis was performed using IBM SPSS Statistics, Version 23.0, software (IBM Corp., Armonk, NY, USA). Chi-square tests were performed to compare the frequency and average value of vitamin D deficiency based on the participants’ general characteristics. To interpret Cramer’s V, the following approach is used: (1) V ∈ (0.1–0.3): weak association, (2) V ∈ (0.4–0.5): medium association, and (3) V > 0.5: strong association [27]. Logistic regression analysis was performed to determine whether the participants’ characteristics affected vitamin D deficiency. We also performed logistic regression analysis to investigate the effect of regular physical activity on vitamin D deficiency by constructing the following sequential models: Model 1 included the frequency of participation in each regular physical activity, Model 2 was adjusted for sex and BMI, and Model 3 included the variables adjusted for in Model 2 together with nutritional education and diet. The significance level was set at *p* < 0.05 for all tests. A power analysis was conducted using G* Power software (G* Power 3.1.7, Heinrich-Heine-University, Düsseldorf, Germany), which indicated that 119 participants were required to attain a power of 0.95 in this study, with an effect size f^2^ = 0.15 and an α = 0.05.

## 3. Results

The general characteristics of the participants with and without vitamin D deficiency were analyzed (Table 1). Of 2811 adolescents analyzed, 2237 (79.6%) were vitamin D deficient. The vitamin D deficiency rate in high school students was higher than that in middle school students (*p* < 0.001) and higher in girls compared to boys (*p* < 0.001). The incidence of vitamin D deficiency was significantly lower in participants who performed intense physical activity (*p* < 0.001), moderate physical activity (*p* = 0.002), and muscular exercise (*p* < 0.001) than in those who did not participate in any activity. However, there were no significant differences between groups in walking status, BMI, nutritional education, and diet status (*p* > 0.05).

Table 2 shows factors associated with vitamin D deficiency, comparing those in the group with vitamin D deficiency to those in the group without vitamin D deficiency. Being in high school (odds ratio (OR) 1.70 (95% confidence interval (CI) 1.40–2.06); *p* < 0.001) and being female (OR 1.43 (95% CI 1.18–1.72); *p* < 0.001) were significantly associated with vitamin D deficiency. Those who did not participate in physical activity (OR 1.56 (95% CI 1.23–2.01); *p* < 0.001) exhibited higher vitamin D deficiency rates than those in the 4–7 days exercise group. For the moderate physical activity variable, those who did not participate in physical activity (OR 1.47 (95% CI 1.15–1.90); *p* = 0.003) exhibited higher vitamin D deficiency levels than those in the 4–7 days group. Vitamin D deficiency rates were also significantly associated with those who did not participate (OR 1.40 (95% CI 1.06–1.85); *p* = 0.018) in muscular exercise compared to those who participated for 4–7 days of the week. For the BMI variable, the group with obesity (OR 1.52 (95% CI 1.09–2.12); *p* = 0.013) showed higher vitamin D deficiency levels than the normal weight group. However, there was no significant association with walking days, nutritional education, and diet status (*p* > 0.05).

Table 3 shows the correlation between participation frequency and vitamin D deficiency risk by physical activity. The risk of vitamin D deficiency in the no exercise participation subgroup of the intense physical activity variable showed an OR of 1.56 (95% CI 1.23–2.01; *p* < 0.001) in Model 1. Model 2 added sex and BMI for analysis and revealed a 1.42-fold increase in vitamin D deficiency risk (OR 1.42 (95% CI 1.10–1.84); *p* = 0.007). Model 3 added nutritional education and diet status for analysis and exhibited a 1.40-fold increase in vitamin D deficiency risk (OR 1.40 (95% CI 1.09–1.81); *p* = 0.010). In the moderate physical activity variable, the risk of vitamin D deficiency in the non-participating subgroup showed an OR of 1.47 (95% CI 1.15–1.90; *p* = 0.003) in Model 1. Model 2 and 3 showed the risk to be 1.39 (OR 1.39 (95% CI 1.07–1.80); *p* = 0.013) and 1.37 times higher (OR 1.37 (95% CI 1.06–1.78); *p* = 0.017), respectively. In the walking days variable, Models 1, 2, and 3 showed no significant changes in vitamin D deficiency risk (*p* > 0.05). For the muscular exercise days variable, the risk of vitamin D deficiency in the non-participating subgroup showed an OR of 1.40 (95% CI 1.06–1.85; *p* = 0.018) in Model 1.

## 4. Discussion

This study aimed to investigate the relationship between vitamin D status and deficiency-related physical activity in adolescents using data from the KNHANES. Although the numbers vary slightly between countries, it is common for many adolescents to exhibit vitamin D deficiency [28]. Differences in vitamin D concentrations by age are poorly reported in Europe and North America; however, lower vitamin D concentrations have been reported in children and adolescents than in adults and older people in the Asia-Pacific region [29]. Domestic studies have reported a significant reduction in vitamin D concentrations and increased deficiency rates with age. In this study, 79.6% of adolescents were vitamin D deficient, and the risk was 1.70-fold higher among high school students than among students in middle school.

25(OH)D is an active type of vitamin D that regulates cell function in multiple ways by controlling more than 200 genes—including cell proliferation, differentiation, apoptosis, and angiogenesis-related genes—as well as calcium homeostasis and immunomodulatory functions [6,30]. Vitamin D is essential for bone health and growth in adolescence. Nevertheless, the results of a study by Lee et al. [31] suggested that lower vitamin D concentrations and a significant increase in the deficiency rate as grade levels increase in Korean youths are linked to factors such as decreased outdoor activities due to Korea’s unique educational environment. Physical education is not included in the college entrance exams and only takes up a minimal part of required education for adolescents [32]. Low serum vitamin D concentration is associated with an increased risk of rickets and osteoporosis and an increased level of osteocalcin [33]. Low serum vitamin D levels are also associated with obesity, cardiovascular disease, idiopathic male fertility, premature ejaculation, and numerous other diseases, such as cancer and diabetes [34,35,36].

Physical activity using muscle contraction is a practical way to increase vitamin D levels. According to a study by Chomistek et al. [37], participating in intense exercise such as running, jogging, basketball, or soccer for longer than 3 h per week can influence high-density lipoprotein cholesterol levels and increase vitamin D levels. This is because outdoor physical activity in sunlight promotes vitamin D synthesis through the interaction between ultraviolet rays and 7-dehydrocholesterol in the skin [38]. Scott et al. [24] confirmed the association between changes in vitamin D concentration and physical activity in older people with sarcopenia through indoor physical activity regardless of sun exposure. Indoor physical activity reportedly improves serum vitamin D levels effectively due to the significant association between vitamin D concentration and both muscle function [39] and exercise performance ability [40].

Vitamin D levels are also associated with body fat percentage and visceral fat in healthy adolescents. However, excessive fat tissue in people with obesity absorbs vitamin D and reduces its availability in the body, synthesis in the skin, and absorption in the intestine [41,42]. In this study, the correlation between the frequency of participation in any type of physical activity and the risk of vitamin D deficiency was observed in intense physical activity, medium-intensity physical activity, and muscle exercise. The lower the frequency of participation in each physical activity, the higher the risk of vitamin D deficiency. When age, sex, and BMI were corrected for in Model 2 and age, sex, BMI, and the presence or absence of nutrition education and a dietary regimen in Model 3, the risk tended to decrease.

In addition to environmental factors, several studies have reported that dietary vitamin D intake positively correlates with serum vitamin D concentration [43]. A balanced diet is preferred because vitamin D-rich foods are assimilated better than supplements [44]. However, if these conditions cannot be met, children and adolescents are recommended to ingest 400 IU supplementary vitamin D per day [31]. Vitamin D plays an important role in the maturity of the immune system in adolescents; therefore, sufficient efforts to balance and maintain vitamin D levels are required since vitamin D is an essential factor in health promotion behaviors and habits. In this study, vitamin D deficiency in adolescence was confirmed to be significantly associated with the frequency of participation in physical activity.

This study is limited by its cross-sectional design; however, this is the first study to utilize the KNHANES data of blood vitamin D levels measured from 1998 to the present. The statistical data summarized changes in the Korean population’s health behavior and chronic diseases from 2008 to 2014. Therefore, this study was conducted using data representing vitamin D levels in Korea despite the limitation of the cross-sectional design; the association between dietary patterns and vitamin D intake must be assessed in the future. The current survey does not include questions regarding the duration of supplement use, indoor and outdoor activity, and whether the students received nutritional education at school. Including these questions will increase the quality of future studies. Future studies will also benefit from including overweight participants and physical activity intensity as variables in data analysis. In addition, to determine the domestic situation of high rates of vitamin D deficiency, vitamin D tests should be included in school check-ups to manage vitamin D concentrations in children and adolescents, and standards for vitamin D concentrations should be established according to age. To increase vitamin D concentration, it is important to increase outdoor activity time to absorb plenty of sunlight; however, it is also necessary to prepare guidelines for supplementation through vitamin D-rich foods or vitamin D supplements during winter. Recognition of the importance of vitamin D sufficiency in overall school health policy and implementation of the educational policy is crucial, including the provision of vitamin D-enhanced foods in school meals. In future studies, it will be necessary to identify these physiological or nutritional factors and consider the variables assessed in this study.

## 5. Conclusions

This study identified the risk factors among age, sex, body fat, nutrition education, and diet status related to physical activity and health factors by determining serum vitamin D concentration in Korean adolescents aged 13–18 years. We used data from the fourth and fifth stages of the KNHANES, between 2008 and 2014. In total, 79.6% of adolescents were vitamin D deficient. The risk and severity were higher in female students than in male students. Regarding vitamin D deficiency and health-related factors, the lower the frequency of participation in each physical activity type, the higher the risk of vitamin D deficiency, and the risk increases significantly with an increase in BMI. However, the participants’ nutritional education and diet status did not significantly affect their risk of vitamin D deficiency. Regarding the risk of vitamin D deficiency according to the frequency of participation in different types of physical activity, the risk was high when frequency of participation was low, and it was lowered when adjustments were made for age, sex, BMI, nutrition education status, and diet status.

## Figures and Tables

**Table 1:** Baseline characteristics of the study sample.

Characteristics		Total	With Vitamin D Deficiency (<20 ng/mL)	Without Vitamin D Deficiency (≥20 ng/mL)	Cramer V Coefficient	*F*	*p*-Value
Variable	Category						
Age	Middle	1632 (58.1)	1242 (76.1)	390 (23.9)	0.001	216.730	<0.001 ***
	High	1179 (41.9)	955 (84.4)	184 (15.6)			
Sex	Male	1484 (52.8)	1141 (76.9)	343 (23.1)	0.001	144.418	<0.001 ***
	Female	1327 (47.2)	1096 (82.6)	231 (17.4)			
Intense physical activity	Never	1089 (38.7)	906 (83.2)	183 (16.8)	0.016	0.508	<0.001 ***
	1–3 days	1113 (39.6)	869 (78.1)	244 (21.9)			
	4–7 days	609 (21.7)	462 (75.9)	147 (24.1)			
Moderate physical activity	Never	1297 (46.1)	1061 (81.8)	236 (18.2)	0.005	1.553	0.002 **
	1–3 days	1040 (37.0)	819 (78.8)	221 (21.3)			
	4–7 days	474 (16.9)	357 (75.3)	117 (24.7)			
Walking days	Never	138 (4.9)	116 (84.1)	22 (15.9)	0.002	13.742	0.423
	1–3 days	435 (15.5)	343 (78.9)	92 (21.1)			
	4–7 days	2238 (79.6)	1778 (79.4)	460 (20.6)			
Muscular exercise days	Never	1633 (58.1)	1351 (82.7)	282 (17.3)	0.001	4.856	<0.001 ***
	1–3 days	815 (29.0)	605 (74.2)	210 (25.8)			
	4–7 days	363 (12.9)	281 (77.4)	82 (22.6)			
Body mass index	Normal	1331 (47.3)	1058 (79.5)	273 (20.5)	0.026	9.539	0.057
	Underweight	838 (29.8)	650 (77.6)	188 (22.4)			
	Overweight	304 (10.8)	240 (78.9)	64 (21.1)			
	Obesity	338 (12.0)	289 (85.5)	49 (14.5)			
Nutritional education	Yes	485 (17.3)	372 (76.7)	113 (23.3)	0.072	9.042	0.097
	No	2326 (82.7)	1865 (80.2)	461 (19.8)			
Diet	Yes	340 (12.1)	275 (80.9)	65 (19.1)	0.589	1.559	0.525
	No	2471 (87.9)	1962 (79.4)	509 (20.6)			

Data are presented as n (%). ** *p* < 0.01, *** *p* < 0.001; tested using the chi-square.

**Table 2:** Odds ratio (95% confidence interval) for vitamin D level and baseline variables.

Characteristics		Odds Ratio (95% Confidence Interval)	*p*-Value
Variable	Category		
Age	Middle	Reference	
	High	1.70 (1.40–2.06)	<0.001 ***
Sex	Male	Reference	
	Female	1.43 (1.18–1.72)	<0.001 ***
Intense physical activity	4–7 day	Reference	
	1–3 day	1.13 (0.90–1.43)	0.294
	Never	1.56 (1.23–2.01)	<0.001 ***
Moderate physical activity	4–7 day	Reference	
	1–3 day	1.22 (0.94–1.60)	0.137
	Never	1.47 (1.15–1.90)	0.003 **
Walking days	4–7 day	Reference	
	1–3 day	0.97 (0.75–1.24)	0.779
	Never	1.36 (0.86–2.18)	0.193
Muscular exercise days	4–7 day	Reference	
	1–3 day	0.84 (0.63–1.13)	0.244
	Never	1.40 (1.06–1.85)	0.018 *
Body mass index	Normal	Reference	
	Underweight	0.89 (0.72–1.10)	0.286
	Overweight	0.97 (0.71–1,31)	0.833
	Obesity	1.52 (1.09–2.12)	0.013 *
Nutritional education	Yes	Reference	
	No	1.23 (0.97–1.55)	0.084
Diet status	Yes	Reference	
	No	0.91 (0.68–1.21)	0.525

* *p* < 0.05, ** *p* < 0.01, *** *p* < 0.001; tested using logistic regression analysis.

**Table 3:** Odds ratio (95% confidence interval) of physical activity and vitamin D deficiency risk.

Characteristics		Model 1		Model 2		Model 3	
Variable	Category	OR (95% CI)	*p*-Value	OR (95% CI)	*p*-Value	OR (95% CI)	*p*-Value
Intense physical activity	4–7 day	Reference		Reference		Reference	
	1–3 day	1.13 (0.90–1.43)	0.294	1.10 (0.87–1.40)	0.422	1.10 (0.863–1.40)	0.445
	Never	1.56 (1.23–2.01)	<0.001 ***	1.42 (1.10–1.83)	0.007 **	1.40 (1.09–1.81)	0.010 **
	*p* for trend	<0.001 ***		0.004 **		0.005 **	
Moderate physical activity	4–7 day	Reference		Reference		Reference	
	1–3 day	1.22 (0.94–1.60)	0.137	1.22 (0.94–1.58)	0.143	1.21 (0.93–1.57)	0.153
	Never	1.47 (1.15–1.90)	0.003 **	1.39 (1.07–1.80)	0.013 *	1.37 (1.06–1.78)	0.017 *
	*p* for trend	0.002 **		0.009 **		0.012 *	
Walkingdays	4–7 day	Reference		Reference		Reference	
	1–3 day	0.97 (0.75–1.24)	0.779	0.92 (0.71–1.19)	0.525	0.92 (0.71–1.19)	0.524
	Never	1.36 (0.86–2.18)	0.193	1.38 (0.86–2.20)	0.184	1.36 (0.85–2.17)	0.204
	*p* for trend	0.423		0.562		0.599	
Muscular exercise days	4–7 day	Reference		Reference		Reference	
	1–3 day	0.84 (0.63–1.13)	0.244	0.86 (0.64–1.16)	0.331	0.86 (0.64–1.16)	0.313
	Never	1.40 (1.06–1.85)	0.018 *	1.33 (0.99–1.78)	0.056	1.31 (0.98–1.75)	0.072
	*p* for trend	<0.001 ***		<0.001 ***		<0.001 ***	

OR, odds ratio; CI, confidence interval; * *p* < 0.05, ** *p* < 0.01, *** *p* < 0.001; tested using multiple logistic regression analysis. Model 1: adjusted for regular exercise (never, <1–3 days, and <4–7 days per week). Model 2: further adjusted for age (middle: 13–15 years and high: 16–18 years), sex, and obesity (BMI < 18.5 kg/m^2^, 18.5–22.9 kg/m^2^, 23–24.9 kg/m^2^, and ≥25 kg/m^2^). Model 3: further adjusted for nutritional factors (education and dietary regimen, yes or no).

## Data Availability

The data presented in this study are available upon request from the authors. Some variables are restricted to preserve the anonymity of the study participants.
